# Why decision curves cross treat-all and treat-none: A PPV and calibration perspective

**DOI:** 10.1016/j.gloepi.2026.100290

**Published:** 2026-10-08

**Authors:** Linard Hoessly

**Affiliations:** Universitätsspital Basel, Switzerland

**Keywords:** 62C05, 62F07, 62H30, 62P10, Net benefit, Positive predictive value, Decision curve analysis, Binary classification, Predictive modelling, Diagnostic accuracy

## Abstract

Net benefit is widely used to assess whether clinical prediction models can improve decision-making, but its interpretation is often difficult in practice. In this note, we show how decision curves can be understood through two familiar quantities: positive predictive value and observed event rates in threshold-defined patient groups. We show that a model has positive net benefit compared with treating no one when, among patients classified as positive, the observed event rate exceeds the decision threshold. Similarly, a model outperforms treating everyone when, among patients classified as negative, the observed event rate is below the decision threshold. These results connect decision-curve performance directly to interpretable forms of threshold-specific calibration. We also describe how positive predictive value curves can complement decision curves by showing, at each threshold, the observed event rate among patients selected for action. Together, these perspectives help explain why a prediction model performs better or worse than default strategies and clarify how decision curve comparisons relate to familiar epidemiological quantities.

## Introduction

Decision-analytic measures play a central role in evaluating the impact of clinical prediction models [1], [2]. In the following, we focus on net benefit (NB), which folds utility considerations directly into the choice of threshold [3], [4] rather than requiring these to be estimated separately, as in explicit utility analysis [5]. This made NB widely used [6]. In particular, while earlier reporting guidelines like TRIPOD [7] mention NB implicitly, more recent ones like TRIPOD-Cluster [8], TRIPOD+AI [9], or TRIPOD-SRMA [10] explicitly recommend, or at least mention, decision curve analysis and NB. Prediction-model validation and reporting, as formalised in the TRIPOD guidelines above, is itself epidemiological work, with positive predictive value (PPV) being standard tools for evaluating diagnostic and screening tests and calibration a standard aspect for probabilistic prediction models. In practice, NB is typically compared with the default strategies treat-all and treat-none for both prognostic and diagnostic models, besides the comparison of different models .

Despite extensive methodological work, tutorials, and applied use [3], [4], [6], [11], [12], [13], [14], [15], interpreting the magnitude of NB can still be difficult [14]. A separate issue is that it is often unclear what it means for the predictions of the underlying model when the NB falls below treat-none or treat-all at a given threshold. In this paper, we address this question by re-expressing these comparisons in terms of PPV and threshold-specific observed risk. Importantly, this re-expression is a diagnostic one: it shows that underperformance relative to treat-all or treat-none is equivalent to a specific, identifiable pattern of threshold-specific miscalibration in the corresponding subgroup. This distinction matters in practice, since miscalibration can often be corrected through recalibration or statistical updating without redeveloping the model [16], whereas improving discrimination typically requires better predictors or a new model and is considerably harder to achieve.

Note that NB is proper as a scoring rule [17]: in expectation, it is maximised by the true risks (i.e., perfect predictions) and cannot be improved upon [18]. Like other evaluation measures it is the case that what we observe in practice is not the expectation but observed values. Hence observed NB is a realisation of a random variable and is therefore subject to sampling variability [19], [20]. For this purpose, it is advisable to display uncertainty, for example via bootstrap confidence bands or, at minimum, threshold-specific uncertainty summaries [1], [21]. NB is analytically linked to PPV [22]. Simulation studies have shown that systematic miscalibration can substantially reduce net benefit [11], which is consistent with the properness of the underlying scoring-rule formulation [18].

Motivated by the practical difficulty of interpreting NB, we revisit a known algebraic link between NB and PPV and use it to provide a intuitive explanation of decision-curve comparisons. We also develop a threshold-specific calibration interpretation of comparisons with the default strategies of treat-none and treat-all. Specifically, we show that a model outperforms treat-none at a given threshold t when the observed event rate among patients classified as positive exceeds that threshold t, and that it outperforms treat-all when the observed event rate among patients classified as negative is below that threshold t. These equivalences translate decision-analytic statements into quantities that are often directly interpretable. Finally, we propose PPV curves as a practical visual companion to decision curves. We do not introduce a new decision metric; rather, we offer a didactic framework and diagnostic visualisation to make net benefit easier to interpret, explain, and troubleshoot in applied clinical prediction research.

### The prediction setting and classification evaluation

Consider n patients. For patient i, a model predicts an event risk $p_{i}\in \left[0,1\right]$, and we observe the outcome $y_{i}\in \left\{0,1\right\}$. The true (unknown) risk is $q_{i}\in \left[0,1\right]$, with perfect prediction given by $p_{i}=q_{i}$ [23], [24].

Given a decision threshold t, patients with $p_{i}\geq t$ are classified as positive and those with $p_{i}<t$ as negative, yielding counts of true positives TP(t), true negatives TN(t), false positives FP(t), and false negatives FN(t). Classification performance is available as (TP(t),FP(t),TN(t),FN(t)). However, most popular classification evaluation measures consist of only one value summarising a specific aspect of performance [25]. Note that in a dataset the numbers of events and non-events are fixed,

(1) $$TP\left(t\right)+FN\left(t\right)=n_{1},\;FP\left(t\right)+TN\left(t\right)=n_{0},$$

where we denote the event fraction by $\pi =\frac{n_{1}}{n}$ and the selection rate by $s_{t}≔\left(TP\left(t\right)+FP\left(t\right)\right)/n$.

#### Net benefit

The NB at threshold t is then defined as [4]

(2) $$NB\left(t\right)\;=\;\frac{TP\left(t\right)}{n}-\frac{FP\left(t\right)}{n}\cdot \frac{t}{1-t},\;t\in \left(0,1\right).$$

This definition can be interpreted as the proportion of the population that are true positives, minus a weighted proportion that are false positives, where the weight t/(1−t) reflects the relative harm of a false positive compared to the benefit of a true positive at threshold t. We next give the definition of NB of treat-none and treat all. Intuitively, as NB is defined by the treated, treating no one gives zero harm and zero benefit, so $NB_{\mathrm{none}}\left(t\right)=0$. Correspondingly, $NB_{\mathrm{all}}\left(t\right)=\pi -\left(1-\pi \right)\frac{t}{1-t}$ which as we treat everyone follows from $TP\left(t\right)=n_{1}$ and $\frac{n_{1}}{n}=\pi$ and $FP\left(t\right)=n-n_{1}$ correspondingly. Note that $NB_{\mathrm{all}}\left(t\right)=\frac{\pi -t}{1-t}$ vanishes at t=π and is positive for t<π and negative for t>π, so treat-all has the higher net benefit below the prevalence and is below treat-none above it.

In medicine, prediction models are typically evaluated over a restricted range of thresholds corresponding to clinically plausible decisions [26]. In this range, comparisons are commonly made against treat-all and treat-none [3]. However, when an established model is already in use, the more relevant benchmark is the comparison to that model rather than the default strategies. As a word of caution, NB is a utility calculation that becomes arbitrary whenever the threshold t is not anchored to real harms/benefits for the specific action and setting.

#### PPV and PPV curves

PPV is the probability that a patient with a positive test truly has the disease, i.e., the proportion of true positives among all positives. In the machine learning literature it is also called precision [25]. In practice, PPV indicates how much confidence a clinician or epidemiologist can place in a positive test, making it central to the evaluation of diagnostic tools and treatment decisions [27]. As in the case of NB curves, we consider PPV curves where we have a curve along (PPV(t),t), where

$$PPV\left(t\right)≔\left\{\begin{array}{cc} \frac{TP\left(t\right)}{TP\left(t\right)+FP\left(t\right)}\;\; & \text{if}TP\left(t\right)+FP\left(t\right)\neq 0\\ 0\; & \text{otherwise}, \end{array}\right)\;t\in \left(0,1\right)$$

#### Calibration

Calibration measures assess how well predicted probabilities agree with observed event rates in a given sample. Such measures are empirical checks and should be distinguished from calibration as a theoretical property of the predictions, for which a hierarchy of increasingly strong definitions exists [28]. A commonly checked form is calibration-in-the-large, which checks agreement between the average predicted risk and the average observed event rate over the whole sample, $\overset{¯}{Y}\approx \overset{¯}{p}$ [1]. Various other methods exist to evaluate calibration, e.g., calibration intercept and slope or calibration curves [1].

Denote by ${\overset{¯}{Y}}_{\geq t}$ and ${\overset{¯}{Y}}_{<t}$ the observed event rate among individuals whose predicted probability is at least t and below t. We can then ask whether these subgroups are themselves calibrated in the sense that the average observed event rate within each group agrees with the corresponding average predicted risk within that group. These questions relate directly to the evaluation of NB as we will see (see Table 1).

### Summary of the main equivalences

**Table 1:** Practical connections between net benefit, threshold-specific event rates, and PPV. Here ${\overset{¯}{Y}}_{\geq t}$ denotes the observed event rate among patients with predicted risk p≥t, and ${\overset{¯}{Y}}_{<t}$ the observed event rate among patients with p<t.

Question	Equivalent condition	Interpretation
Is the model better than treat-none at threshold t?	${\overset{¯}{Y}}_{\geq t}>t$	Among patients classified positive, the observed event rate must exceed the treatment threshold. Otherwise, the flagged group is not high-risk enough to justify intervention relative to treating nobody.
Is the model better than treat-all at threshold t?	${\overset{¯}{Y}}_{<t}<t$	Among patients classified negative, the observed event rate must be below the treatment threshold. Otherwise, withholding intervention from this group is not justified.
How do PPV curves help interpret decision curves?	$\mathrm{PPV}\left(t\right)={\overset{¯}{Y}}_{\geq t}$	PPV at threshold t describes the following: among patients flagged positive, what fraction truly has the outcome.
What does poor decision-curve performance mean?	${\overset{¯}{Y}}_{\geq t}\leq t$ or ${\overset{¯}{Y}}_{<t}\geq t$	Falling below treat-none or treat-all at t has a direct interpretation in terms of threshold miscalibration at t.

### Related literature

Several introductions and tutorials explain how to use and interpret NB and decision curve analysis in applied clinical research [14], [15], [29], [30], [31]. Our focus is different: we aim to make comparisons with treat-none and treat-all easier to interpret by expressing them through PPV and threshold-specific observed event rates.

The algebraic link between NB and PPV has been noted previously [22, p. 12]. Here, we use this link to give explicit conditions under which a model outperforms the default strategies in terms of PPV. The effect of miscalibration on NB has also been studied through simulations [11], and it was shown that moderate calibration guarantees, in expectation, NB at least as high as the default strategies of treat-none and treat-all [28](termed *non-harmful decision making* in [28]). We complement this by showing that moderate calibration is sufficient but not necessary for this guarantee, and commenting on other aspects of moderate calibration. In particular, our results are threshold-specific empirical conditions which explain, at each threshold, why a decision curve lies above or below the default strategies.

Finally, we propose PPV curves as a visual companion to decision curves. Related displays include classification plots, which show true-positive and true-negative rates across thresholds [15], [32], and lift charts, which summarise enrichment among patients ranked by predicted risk [33]. In contrast, PPV curves show the observed event rate among patients classified as positive at each decision threshold, giving a direct visual explanation of the corresponding decision-curve results.

## Understanding fundamental conditions for net benefit through PPV and calibration

### Interpreting net benefit through PPV curves

The following formula expresses PPV through NB and $s_{t}$ [22]

(3) $$PPV\left(t\right)=\left\{\begin{array}{cc} \;\frac{n\;NB\left(t\right)}{TP\left(t\right)+FP\left(t\right)}\left(1-t\right)+t\;\; & \text{if}TP\left(t\right)+FP\left(t\right)\neq 0\\ 0\; & \text{otherwise}, \end{array}\right)\;t\in \left(0,1\right).$$

In words, this formula PPV through NB: it says that PPV is the threshold t plus a correction term proportional to NB times $1/s_{t}=\frac{n}{TP\left(t\right)+FP\left(t\right)}$, giving a direct way to translate between the two quantities.

This identity links two perspectives: the decision-analytic view through net benefit and the predictive view through PPV. Although there is no one-to-one correspondence between NB(t) and PPV(t), they each place constraints on the other, as outlined for PPV in Appendix A. In what follows, we explore how this relationship can be used to derive equivalences between model comparisons to treat all and treat none (detailed mathematical derivations are in Appendix A.1). These can easily be visualised along PPV curves as in Section “Example visualisation of PPV curves”, enabling direct visual complements of NB comparisons.

• NB(t) **better than treat-none means the proportion of true positives among all positives is bigger than t**, t∈(0,1):
  (4) NB(t)>0⟺PPV(t)>t.
  Correspondingly, a model is better than treat-none if, among those it flags positive, the PPV(t) exceeds the threshold itself. For instance, at t=0.1, the model must achieve PPV(t)>0.1 for positive NB.
• NB(t) **outperforms treat-all at** t, t∈(0,1):
  (5) $$NB\left(t\right)>\pi -\left(1-\pi \right)\frac{t}{1-t}\;⟺PPV\left(t\right)>\frac{\pi -t}{s_{t}}+t.$$
  Compared with (4), the bar for PPV(t) is raised by $\left(\pi -t\right)/s_{t}$. This margin is positive for t<π, where treat-all is the harder comparator, and negative for t>π, where clearing the treat-none bar already suffices.

### Interpreting net benefit through decision-threshold calibration

In this section, we derive equivalences between the threshold calibration checks through observed event rates introduced in Section “Calibration” and NB comparisons to treat all and treat none.

Let ${\overset{¯}{Y}}_{\geq t}$ denote the observed event rate among individuals whose predicted probability exceeds or equals t, ${\overset{¯}{Y}}_{<t}$ the observed event rate among those whose predicted probability is below t and let ${\overset{¯}{p}}_{\geq t}$, ${\overset{¯}{p}}_{<t}$ denote the average predicted probabilities over the corresponding groups. If the two groups defined by t are calibrated in the sample sense of Section “Calibration”, i.e.

$${\overset{¯}{Y}}_{\geq t}\approx {\overset{¯}{p}}_{\geq t},\;{\overset{¯}{Y}}_{<t}\approx {\overset{¯}{p}}_{<t},$$

then, by definition (except in degenerate cases) ${\overset{¯}{p}}_{\geq t}>t$ and ${\overset{¯}{p}}_{<t}<t$, the observed event rates in the two groups fall on the corresponding sides of t. This motivates two simple decision-threshold calibration checks

(1) ${\overset{¯}{Y}}_{\geq t}>t$
(2) ${\overset{¯}{Y}}_{<t}<t.$

Surprisingly, these are equivalent to the following, where we assume that there are some cases classified positive and negative under threshold t∈(0,1), i.e. $1>s_{t}>0$ (detailed derivations are given in Appendix B)

• NB(t) **better than treat-none means** ${\overset{¯}{Y}}_{\geq t}>t$, **i.e., observed event rate among individuals whose predicted probability is at least** t **is in the right direction:** We can write
  (6) $$NB\left(t\right)=\frac{s_{t}}{1-t}\;\left({\overset{¯}{Y}}_{\geq t}-t\right).$$
  Thus NB(t)>0 exactly when ${\overset{¯}{Y}}_{\geq t}>t$. More generally, net benefit can be viewed as the product of two factors: a calibration surplus $\left({\overset{¯}{Y}}_{\geq t}-t\right)$ and a selection-rate multiplier $s_{t}/\left(1-t\right)$, which scales this surplus according to how many individuals are classified as positive.
• NB(t) **outperforms treat-all at** t **means** ${\overset{¯}{Y}}_{<t}<t$, **i.e., observed event rate among individuals whose predicted probability is below** t **is in the right direction:**
  (7) $$NB\left(t\right)>NB_{\mathrm{all}}\left(t\right)\;⟺\;{\overset{¯}{Y}}_{<t}<t.$$
  A prediction model beats treat all at threshold t exactly when the patients not treated (those with predicted risk below t) truly have an observed event rate below t. In other words, it is beneficial to withhold treatment from the below-threshold group only if that group is indeed low risk in the observed data.

### Example visualisation of PPV curves

To illustrate PPV curves, we present examples of prediction models together with their decision curve analysis (net benefit) and the corresponding PPV curves, complemented with plots of the distribution of predictions. Calibration plots for both examples are given in Appendix C. As the equivalences in Section “Interpreting net benefit through decision-threshold calibration” are analytic, these plots do not provide independent evidence; they display the same crossings in another, familiar form.

Decision curves were produced with the dcurves package [34]. We implemented an analogous routine to compute and plot PPV curves. Each decision curve figure shows the model-specific NB curves as well as the treat-all and treat-none reference curves. Each PPV figure includes at least 3 curves and lines, whose colour is shared across all figures for consistency:

• the treat-none reference (the main diagonal), consistent with (4);
• the PPV curve;
• for each model, a dashed “treat-all comparison” curve (same colour as the model’s PPV curve), obtained from the right-hand side of (5).

Colours are matched across figures: a model’s NB curve, PPV curve, and dashed treat-all comparison curve share the same colour.

**Figure:**
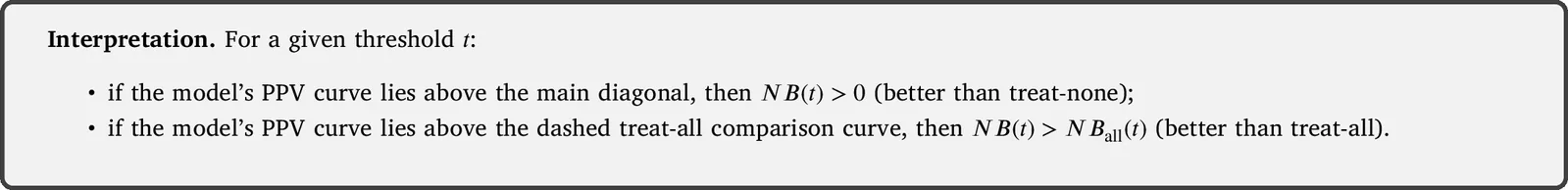

Furthermore we illustrate density plots which shows the distribution of predicted risks. The spread of the predictions shows over which range a model separates patients at all. The proportion above a threshold t is the selection rate $s_{t}$, which is a multiplier in (6). Where this becomes thin, both NB and PPV rest on few observations, and if the density line is not continued after t there are no more predictions above t.

We use the following illustrative examples:

**Fig. 1:**
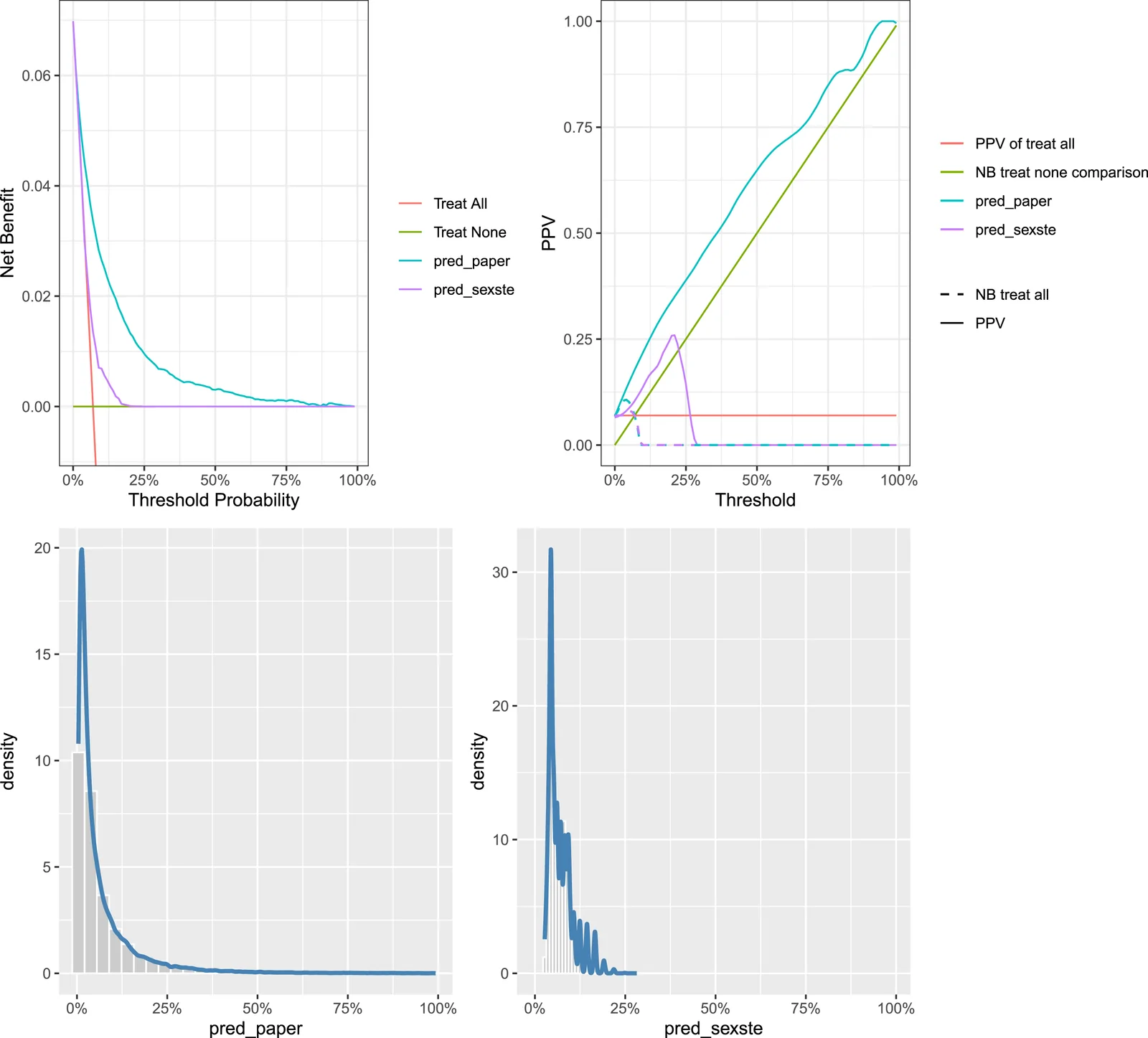
Net benefit curve with corresponding PPV curve for GUSTO-I.

• Fig. 1 (GUSTO-I [35], complete cases): we compare (i) a “published-style” logistic regression including flexible effects for age (ns(age,df=4)) and heart rate (ns(pulse,df=3)), Killip class, winsorised systolic blood pressure (${\text{sysbp}}_{w}=\min\left(\text{sysbp},120\right)$), infarct location (miloc), and an age×Killip interaction; with (ii) a parsimonious logistic regression using sex and ST-elevation burden (ste).
• Fig. 2 (SUPPORT [36], complete cases): we compare (i) a richer logistic regression model including flexible effects for age, mean arterial pressure, and creatinine (restricted cubic splines with 5 knots: rcs(⋅,5)), together with sex, disease class (dzclass), number of comorbidities (num.co), coma score (scoma), white blood cell count (wblc), and respiratory status (resp); with (ii) a simple logistic regression using only age and mean arterial pressure.

The advantages of PPV curves are illustrated in Fig. 1, Fig. 2. Fig. 1 already illustrates some points: above roughly t=0.23 the parsimonious models’ NB curve are visually indistinguishable from zero, while the PPV panel still separates the two curves, with the more complex model’s PPV curve rising towards one and the parsimonious model’s dropping to zero. The latter is the convention PPV(t)=0 when TP(t)+FP(t)=0: above that threshold the parsimonious model flags no patients, as the density plot shows. In the SUPPORT example in Fig. 2, the parsimonious model’s PPV curve lies below the dashed treat-all comparison curve between roughly t=0.10 and t=0.23, so it does not beat treat-all there. It stays above the diagonal until roughly t=0.625, where it again drops to zero because no patients are flagged beyond that point, which can also be observed from the NB curve. The richer model mostly stays above the diagonal and the dashed curve throughout the plotted range. The two panels therefore encode the same comparisons, but the PPV panel states them as an event rate among the flagged patients, which is interpretable directly for a clinician or epidemiologist.

The same crossings admit a calibration reading, as $PPV\left(t\right)={\overset{¯}{Y}}_{\geq t}$. A PPV curve above the diagonal is therefore ${\overset{¯}{Y}}_{\geq t}>t$ by (6), and a PPV curve lying below the dashed comparison curve, as for the parsimonious model in Fig. 2, corresponds to ${\overset{¯}{Y}}_{<t}>t$ by (7): the patients left untreated have an observed event rate above t. The below-threshold rate ${\overset{¯}{Y}}_{<t}$ is not plotted directly in Fig. 1, Fig. 2; it is shown in the lower panels of Fig. A.3, Fig. A.4. In both figures the richer model keeps both subgroup event rates on the correct side of t over a wider threshold range, which is what its higher decision curve records. In that sense, PPV provides clinically relevant information that is not explicit in NB: it is the probability that a patient flagged positive truly has the outcome, displayed at each threshold. Furthermore, at higher thresholds, net benefit curves often shrink towards zero because $s_{t}$ falls, by (6), rather than as an artefact of plot scale. This makes comparisons with treat-all and treat-none harder to read. PPV curves make the same comparisons immediate: the diagonal PPV(t)=t corresponds to the treat-none boundary, and the dashed curve to the treat-all boundary. Wherever a models PPV curve lies above these references, its net benefit is above the corresponding default strategy at that threshold. However, PPV curves do not carry the utility-scaled magnitude of NB itself, and comparing two models PPV curves directly corresponds to comparing their NB only when their selection rates $s_{t}$ agree at that threshold (Appendix D).

R version 4.4.3 was used for the illustration, where we loaded the GUSTO-I [35] and SUPPORT [36] dataset through Hmisc [37].

**Fig. 2:**
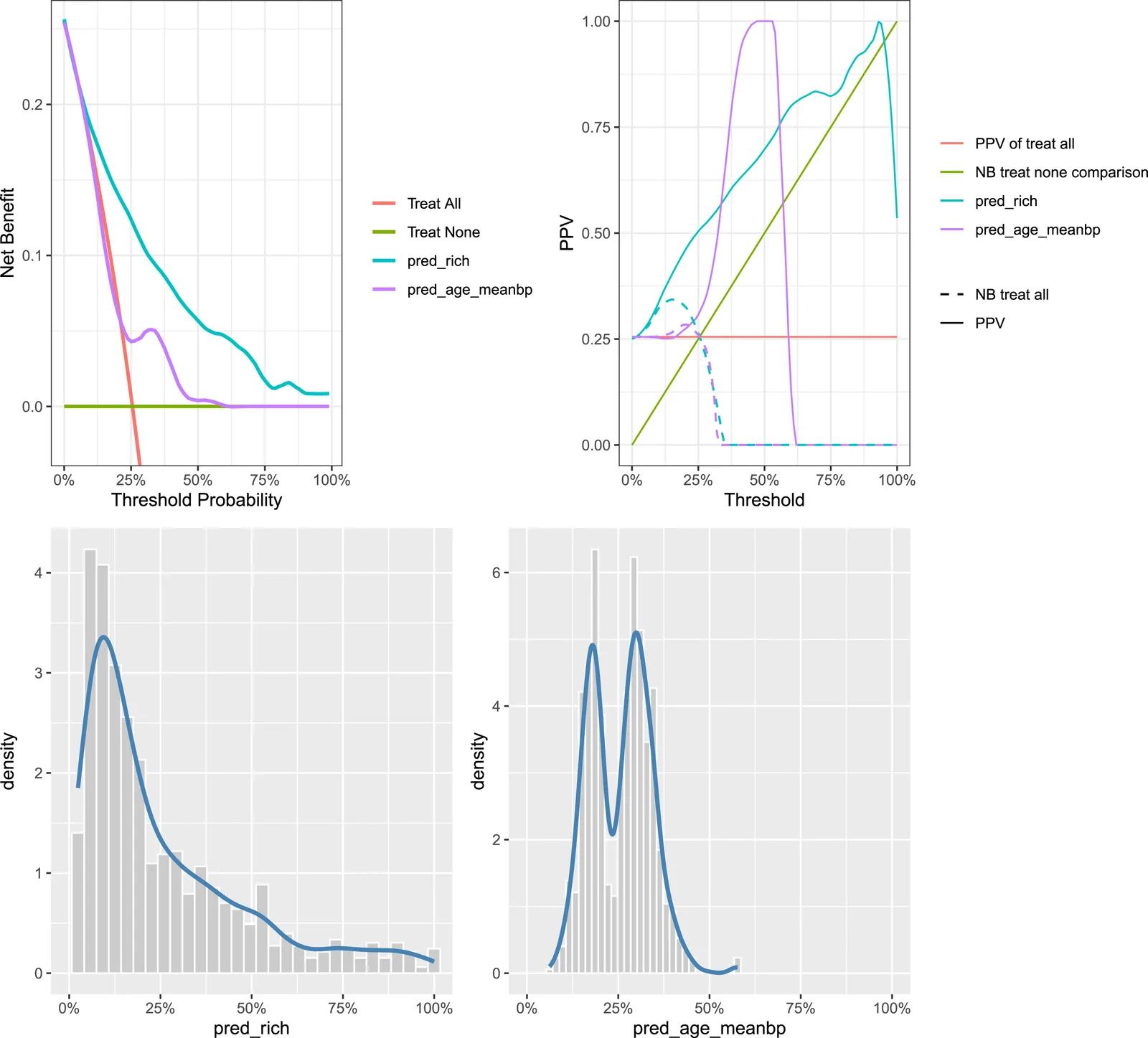
Net benefit curve with corresponding PPV curve for SUPPORT.

### Analytical arguments on calibration and net benefit

We review the observations that miscalibration can substantially reduce clinical utility and may even lead to NB below the treat-all or treat-none strategies [11].Two failure modes were highlighted: systematic overestimation can yield NB(t)<0 for thresholds t>π (worse than treat-none), whereas systematic underestimation can yield $NB\left(t\right)<NB_{\text{all}}\left(t\right)$ for thresholds t<π (worse than treat-all). Both mechanisms operate in expectation: over- and underestimation are properties of the predictions relative to the underlying rates, whereas ${\overset{¯}{Y}}_{\geq t}$ and ${\overset{¯}{Y}}_{<t}$ are observed quantities subject to sampling variability [19], [20]. Miscalibration therefore makes the corresponding inequality more likely to be violated, but neither guarantees a violation in a given sample nor is required for one to appear by chance. Both effects can be explained by the observations in Section “Interpreting net benefit through decision-threshold calibration”. For convenience we briefly go through the arguments below.

**Overestimation.** If risks are systematically overestimated, predicted risks tend to exceed the event rates observed in the corresponding groups. Individuals belonging to groups whose observed event rate is below t are then classified as positive, which dilutes the treated set so that ${\overset{¯}{Y}}_{\geq t}<t$, implying NB(t)<0 by (6). This is more likely for high thresholds, e.g. t>π [11].

**Underestimation.**If risks are systematically underestimated, predicted risks tend to fall below the event rates observed in the corresponding groups. Individuals belonging to groups whose observed event rate is above t are then classified as negative, which inflates the untreated set’s event rate so that ${\overset{¯}{Y}}_{<t}>t$, equivalent to $NB\left(t\right)<NB_{\mathrm{all}}\left(t\right)$ by (7). This is most likely for low thresholds, e.g. t<π, matching the simulations in [11].

In summary, these failures correspond to violations of the calibration inequalities ${\overset{¯}{Y}}_{\geq t}>t$ (treated set) and ${\overset{¯}{Y}}_{<t}<t$ (untreated set), with their impact scaled by $s_{t}$ and $1-s_{t}$, respectively.

For the interested reader in Appendix E we give

• a further decomposition of NB. This can be used to show that for many observations perfect predictions likely lead to positive observed NB(t).
• notes to show how PPV curves can be used to compare NBs of models.

## Reviewing the connection of moderate calibration to NB

### Why does moderate calibration guarantee NB at least as high as the defaults in expectation?

Denote by ${\overset{¯}{Y}}_{i:p_{i}=r}$ the mean over all outcomes where the predictions equal r. Then, moderate calibration as in [28] can be expressed as Follows:For any r∈[0,1] that is predicted at least once it holds that

(8) $$E\left({\overset{¯}{Y}}_{i:p_{i}=r}\right)=r.$$

[28] showed that moderate calibration implies, in expectation, NB at least as high as treat-none and treat-all.

For moderate calibration it follows that if there is a prediction above or equal t, we have

$$E\left({\overset{¯}{Y}}_{\geq t}\right)\geq t,$$

which, from (6), implies that NB is not below treat-none. To see this let $r_{1},\ldots ,r_{k}$ be the k different values predicted above or equal to t for 1>t>0, and with each taken $n_{1},\ldots ,n_{k}$ times. Then we can rewrite the expectation by (8) and the linearity of the expectation as

$$E\left({\overset{¯}{Y}}_{\geq t}\right)=E\left(\frac{\overset{k}{\underset{j=1}{\sum }}n_{j}{\overset{¯}{Y}}_{i:p_{i}=r_{j}}}{\overset{k}{\underset{j=1}{\sum }}n_{j}}\right)=\frac{\overset{k}{\underset{j=1}{\sum }}n_{j}E\left({\overset{¯}{Y}}_{i:p_{i}=r_{j}}\right)}{\overset{k}{\underset{j=1}{\sum }}n_{j}}=\frac{\overset{k}{\underset{j=1}{\sum }}n_{j}r_{j}}{\overset{k}{\underset{j=1}{\sum }}n_{j}}\geq \underset{1\leq j\leq k}{\min}r_{j}\geq t.$$

An analogous argument shows that under moderate calibration $E\left({\overset{¯}{Y}}_{<t}\right)\leq t$, which again by (7) implies that NB is not below treat-all.

However, note that these implications are entirely theoretical as they concern statements that hold in expectation.

### What does this mean in practice?

The equivalence of the calibration inequalities and improvement to treat all and treat none for net benefit in Section “Interpreting net benefit through decision-threshold calibration” are analytic. They hold for observed quantities, and by taking expectations on both sides they can be translated to a theoretical equivalence in expectation. However, we note that

• it is typically impossible to conclude in practice that moderate calibration holds as it is a property in expectation. This is clear since whenever we have numerical variables in prediction models, the variable value combinations often lead to unique predicted probabilities [28].
• observed NB above the default strategies does not imply the same holds in expectation, nor the converse, since what we observe in practice is not the expectation but observed values [19], [20].
• moderate calibration guarantees this only overall; NB can still fall below a default strategy within subgroups. An example is provided in Appendix F.1.
• moderate calibration is sufficient but not necessary for NB to be at least as high as both defaults. An example is provided in Appendix F.2.

## Discussion

PPV, decision-threshold calibration, and NB describe model performance from different but complementary perspectives. In this paper, we have shown that these perspectives are closely linked through comparisons of NB with the default strategies of treat-none and treat-all.

PPV, the probability that a patient classified as positive at a given threshold truly has the outcome, is clinically intuitive and answers the question: “If the model recommends action, how likely is it to be correct?” PPV curves therefore provide a useful companion to decision curves. They preserve the threshold-based clinical interpretation of NB while often making the comparison with default strategies easier to understand. In particular, superiority over treat-none or treat-all can be assessed by whether the PPV curve lies above or below the corresponding threshold-specific reference curve. This may be especially helpful at higher thresholds, where NB curves approach zero because few patients are flagged [11], [15], [31]. By (6) this is a $s_{t}/\left(1-t\right)$ effect rather than a matter of plot scale, and PPV curves show directly whether the flagged group is still enriched relative to t. Decision-threshold calibration provides a second, complementary interpretation. A model performs worse than treat-none or treat-all at threshold t if and only if the observed event rate in the corresponding subgroup is on the wrong side of t, as described in Section “Interpreting net benefit through decision-threshold calibration”. For example, if a model performs worse than treat-none at threshold t, then

$${\overset{¯}{Y}}_{\geq t}<t.$$

That is, among patients with predicted risk at least t, the observed event rate is below the threshold required to justify action. Such deviations may arise from sampling variability [19], [20], but they may also indicate systematic miscalibration and thus motivate recalibration or model updating. Note that we focused on the comparison to treat-none above, but in the same way the comparison to treat all connects to the threshold miscalibration from Section “Interpreting net benefit through decision-threshold calibration”.

Because NB and decision curve analysis are now widely recommended in studies of clinical prediction models [7], [8], [9], [10], we hope these connections make NB easier to interpret and apply. In applied analyses, this suggests: report NB over clinically relevant thresholds, consider adding PPV curves as an explanatory companion, add confidence bands to estimate uncertainty, and inspect threshold-specific observed event rates above and below the threshold when a model performs poorly relative to treat-none or treat-all.

Finally, we note that our focus has been on the NB of the treated, i.e. NB(t) as defined in (2), which counts the true positives gained by treating and charges the false positives which were nonetheless treated. In some settings, however, the dual quantity is more relevant [13]: the NB of the untreated

$$NB_{u}\left(t\right)=\frac{TN\left(t\right)}{n}-\frac{1-t}{t}\cdot \frac{FN\left(t\right)}{n},$$

which counts the true negatives correctly not treated and charges the events missed, at the reciprocal exchange rate (1−t)/t. Parallel results can then be derived using negative predictive value [38] together with decision-threshold calibration, leading to analogous visualisations and interpretations for the untreated group.

## AI use

During the preparation of this manuscript, we used GPT-4o and Claude for minor language edits as well as for latex support aiming to enhance readability, and elicit.com for literature search. After using it, we reviewed and edited the content as needed and take full responsibility for its content.

## Declaration of competing interest

The authors declare that they have no known competing financial interests or personal relationships that could have appeared to influence the work reported in this paper.

## Figures and Tables

**Fig. A.3:**
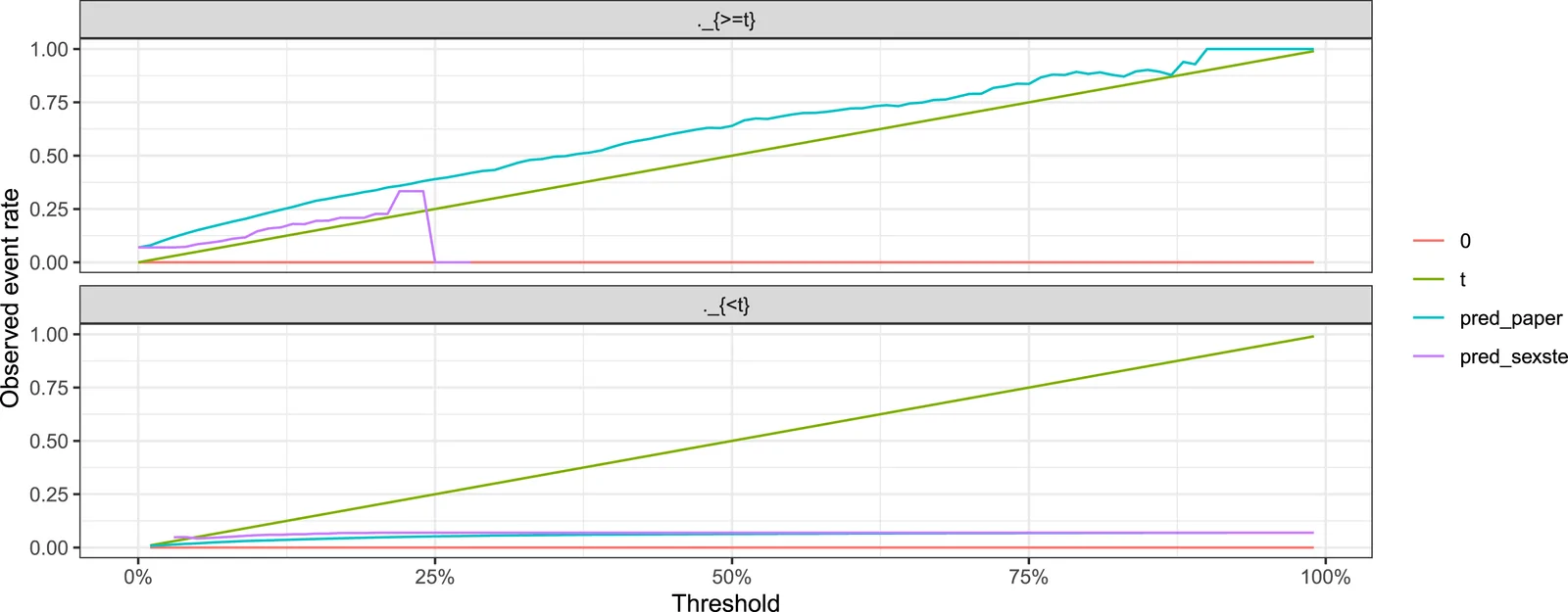
Threshold-specific observed event rates for GUSTO-I.

**Fig. A.4:**
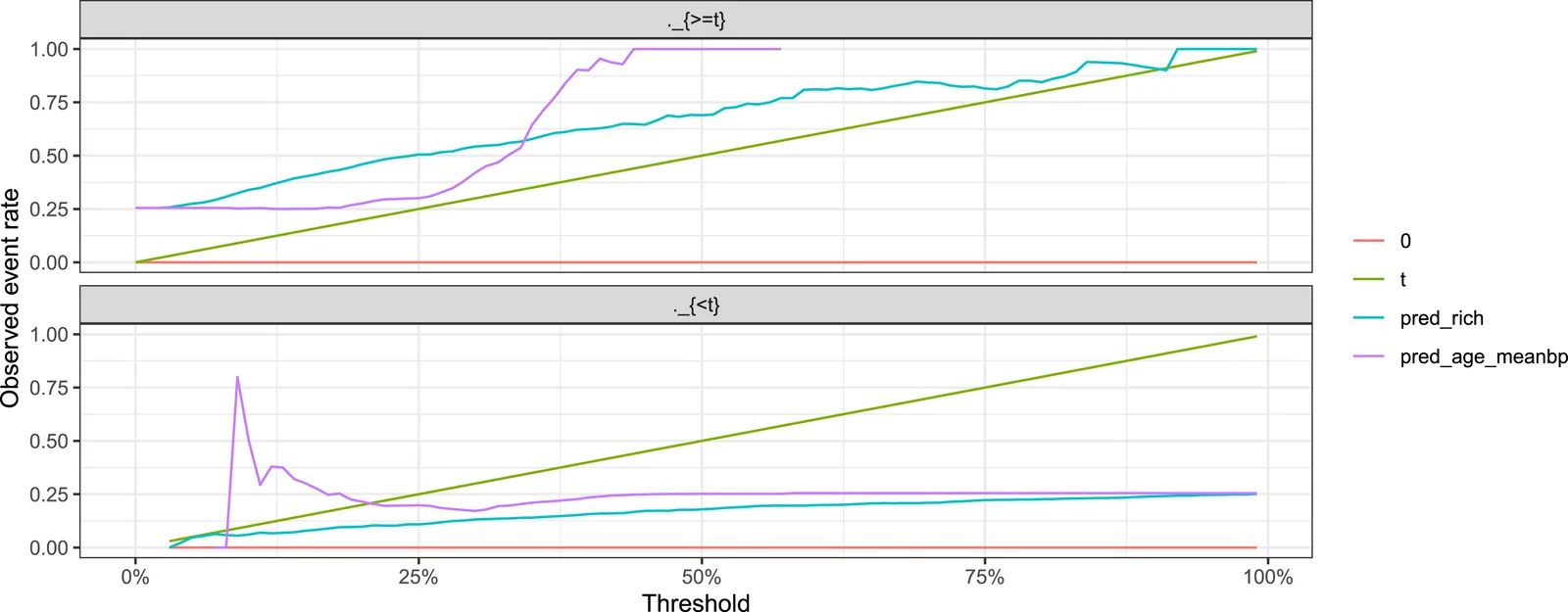
Threshold-specific observed event rates for SUPPORT.

## Appendix A. Bounds on PPV implied by net benefit

For $s_{t}>0$, (3) gives $PPV\left(t\right)=t+\left(1-t\right)\;NB\left(t\right)/s_{t}$. For fixed NB(t), this is decreasing in $s_{t}$ if NB(t)>0 and increasing if NB(t)<0. Since $TP\left(t\right)\leq n_{1}$ and $FP\left(t\right)\leq n_{0}$, the largest selection rate compatible with NB(t) is

$$s_{\max}\left(t\right)=NB\left(t\right)+\min\left\{\frac{\pi -NB\left(t\right)}{t},\;\frac{1-\pi }{1-t}\right\}.$$

With $b\left(t\right)≔t+\left(1-t\right)\;NB\left(t\right)/s_{\max}\left(t\right)$, this yields the sharp bounds

$$PPV\left(t\right)\in \left\{\begin{array}{cc} \left[\;b\left(t\right),\;1\;\right],\; & NB\left(t\right)>0,\\ \left\{0,\;t\right\},\; & NB\left(t\right)=0,\\ \left[\;0,\;b\left(t\right)\;\right],\; & NB\left(t\right)<0. \end{array}\right)$$

Thus NB(t) does not determine PPV(t), but it restricts its feasible range.

### A.1. Outperforming treat all

The net benefit of treating everyone is

$$NB_{\mathrm{all}}\left(t\right)=\pi -\left(1-\pi \right)\frac{t}{1-t},\;\text{with}\pi =\frac{n_{1}}{n}.$$

Using (3), we rewrite

$$NB\left(t\right)-NB_{\mathrm{all}}\left(t\right)=\frac{s_{t}}{1-t}\left(PPV\left(t\right)-t\right)-\left(\pi -\left(1-\pi \right)\frac{t}{1-t}\right)>0$$

by multiplying by (1−t)>0 and rearranging, giving

$$s_{t}\left(PPV\left(t\right)-t\right)>\pi -t\;⟺\;PPV\left(t\right)>\frac{\pi -t}{s_{t}}+t.$$

## Appendix B. Mathematical derivations for calibration

### B.1. Better than treat-none

Let $s_{t}>0$, then ${\overset{¯}{Y}}_{\geq t}≔TP\left(t\right)/\left(TP\left(t\right)+FP\left(t\right)\right)$ and

$$\frac{TP\left(t\right)}{n}=s_{t}\;{\overset{¯}{Y}}_{\geq t},\;\frac{FP\left(t\right)}{n}=s_{t}\;\left(1-{\overset{¯}{Y}}_{\geq t}\right).$$

Substituting into $NB\left(t\right)=\frac{TP\left(t\right)}{n}-\frac{t}{1-t}\frac{FP\left(t\right)}{n}$ yields

$$NB\left(t\right)=s_{t}{\overset{¯}{Y}}_{\geq t}-\frac{t}{1-t}s_{t}\left(1-{\overset{¯}{Y}}_{\geq t}\right)=\frac{s_{t}}{1-t}\left({\overset{¯}{Y}}_{\geq t}-t\right),$$

which proves (6).

### B.2. Outperforming treat all

Let $w≔\frac{t}{1-t}$, t∈(0,1). Using (1) we write

$$NB\left(t\right)-NB_{\mathrm{all}}\left(t\right)=\left(\frac{TP\left(t\right)}{n}-w\frac{FP\left(t\right)}{n}\right)-\left(\frac{n_{1}}{n}-w\frac{n_{0}}{n}\right)=\frac{-FN\left(t\right)+w\;TN\left(t\right)}{n},$$

and

$${\overset{¯}{Y}}_{<t}=\frac{FN\left(t\right)}{TN\left(t\right)+FN\left(t\right)}.$$

As $FN\left(t\right)=n\left(1-s_{t}\right){\overset{¯}{Y}}_{<t}$ and $TN\left(t\right)=n\left(1-s_{t}\right)\left(1-{\overset{¯}{Y}}_{<t}\right)$, we get

$$NB\left(t\right)-NB_{\mathrm{all}}\left(t\right)=\left(1-s_{t}\right)\left[w-\left(1+w\right){\overset{¯}{Y}}_{<t}\right]=\frac{1-s_{t}}{1-t}\;\left(t-{\overset{¯}{Y}}_{<t}\right),$$

as $w=\frac{t}{1-t}$ and $1+w=\frac{1}{1-t}$. Therefore, for $s_{t}<1$,

$$NB\left(t\right)>NB_{\mathrm{all}}\left(t\right)\;⟺\;{\overset{¯}{Y}}_{<t}<t.$$

## Appendix C. Complementing visualisation to equivalence calibration and NB from Section “Interpreting net benefit through decision-threshold calibration” in the settings described in Section “Example visualisation of PPV curves”

See Fig. A.3, Fig. A.4.

## Appendix D. Better models for net benefit in terms of calibration

For each model m∈{1,2}, define

$$s_{m,t}≔\frac{TP_{m}\left(t\right)+FP_{m}\left(t\right)}{n},\;{\overset{¯}{Y}}_{m,\geq t}≔\frac{TP_{m}\left(t\right)}{TP_{m}\left(t\right)+FP_{m}\left(t\right)},\;{\overset{¯}{Y}}_{m,<t}≔\frac{FN_{m}\left(t\right)}{TN_{m}\left(t\right)+FN_{m}\left(t\right)}.$$

Then

$$\frac{TP_{m}\left(t\right)}{n}=s_{m,t}{\overset{¯}{Y}}_{m,\geq t},\;\frac{FP_{m}\left(t\right)}{n}=s_{m,t}\left(1-{\overset{¯}{Y}}_{m,\geq t}\right),$$

and hence

$$NB_{m}\left(t\right)=\frac{TP_{m}\left(t\right)}{n}-\frac{t}{1-t}\frac{FP_{m}\left(t\right)}{n}=\frac{s_{m,t}}{1-t}\;\left({\overset{¯}{Y}}_{m,\geq t}-t\right).$$

Moreover, with prevalence $\pi =n_{1}/n$,

$$\pi =s_{m,t}{\overset{¯}{Y}}_{m,\geq t}+\left(1-s_{m,t}\right){\overset{¯}{Y}}_{m,<t},\;NB_{\mathrm{all}}\left(t\right)=\frac{\pi -t}{1-t},$$

so

$$NB_{m}\left(t\right)=NB_{\mathrm{all}}\left(t\right)+\frac{1-s_{m,t}}{1-t}\;\left(t-{\overset{¯}{Y}}_{m,<t}\right).$$

## Appendix E. Advanced considerations

### E.1. A further decomposition of net benefit

Define the selected-set calibration error

$$\Delta_{t}≔{\overset{¯}{Y}}_{\geq t}-{\overset{¯}{p}}_{\geq t}=PPV\left(t\right)-{\overset{¯}{p}}_{\geq t},$$

where ${\overset{¯}{p}}_{\geq t}$ is the mean predicted risk and ${\overset{¯}{Y}}_{\geq t}$ the observed event rate among those with $\overset{ˆ}{p}\geq t$. Then

$$NB\left(t\right)=\frac{s_{t}}{1-t}\;\left({\overset{¯}{Y}}_{\geq t}-t\right)=\frac{s_{t}}{1-t}\left(\left({\overset{¯}{p}}_{\geq t}-t\right)+\Delta_{t}\right).$$

This decomposes net benefit into

• an enrichment term $\frac{s_{t}}{1-t}\left({\overset{¯}{p}}_{\geq t}-t\right)$, which adds how far the average predicted risk in the selected set is at least t, and
• a calibration term $\frac{s_{t}}{1-t}\Delta_{t}$, which adds a bonus if $\Delta_{t}>0$ and a penalty if $\Delta_{t}<0$.

Hence for a very large number of observations with perfect predictions, leading to a calibration term of roughly zero and positive enrichment means that perfect predictions for net benefit can be expected to be positive by large sample argument (i.e., law of large numbers or central limit theorem [39]).

Also note that the factor $s_{t}/\left(1-t\right)$ implies that miscalibration matters most when many patients are selected (large $s_{t}$) and at lower thresholds.

### E.2. Understanding model comparisons in NB through PPV and calibration

In the following, we derive conditions for a model to be superior in net benefit at threshold t and express them equivalently in terms of PPV and calibration. These interpretations are less direct than those in Section “Understanding fundamental conditions for net benefit through PPV and calibration”, but the PPV-based condition is particularly useful in practice because it can be visualised easily via PPV curves.

#### E.2.1. Comparing two models in NB through PPV

Let models $M_{1}$ and $M_{2}$ be given with their net benefits denoted

$$NB^{\left(j\right)}\left(t\right)=\frac{TP^{\left(j\right)}\left(t\right)}{n}-\frac{t}{1-t}\frac{FP^{\left(j\right)}\left(t\right)}{n},\;j=1,2.$$

Then $M_{1}$ outperforms $M_{2}$ in terms of NB(t) if and only if we have

(9) $$NB^{\left(1\right)}\left(t\right)>NB^{\left(2\right)}\left(t\right)\;⟺\;PPV^{\left(1\right)}\left(t\right)>t+\left(1-t\right)\frac{n\cdot NB^{\left(2\right)}\left(t\right)}{TP^{\left(1\right)}\left(t\right)+FP^{\left(1\right)}\left(t\right)}.$$

Graphically, this means that at each threshold t, $M_{1}$’s PPV curve must lie above the reference curve defined by $M_{2}$’s net benefit, the right hand side of (9).

#### E.2.2. Higher net benefit as a calibration condition

For each model m∈{1,2}, assume that at threshold t∈(0,1) there are both positive and negative classes. Then denote

$$s_{m,t}≔\frac{TP_{m}\left(t\right)+FP_{m}\left(t\right)}{n},\;{\overset{¯}{Y}}_{m,\geq t}≔\frac{TP_{m}\left(t\right)}{TP_{m}\left(t\right)+FP_{m}\left(t\right)},\;{\overset{¯}{Y}}_{m,<t}≔\frac{FN_{m}\left(t\right)}{TN_{m}\left(t\right)+FN_{m}\left(t\right)},$$

from which we can derive the following (see Appendix D),

$$NB_{m}\left(t\right)=\frac{s_{m,t}}{1-t}\;\left({\overset{¯}{Y}}_{m,\geq t}-t\right)\;=\;NB_{\mathrm{all}}\left(t\right)+\frac{1-s_{m,t}}{1-t}\;\left(t-{\overset{¯}{Y}}_{m,<t}\right).$$

Hence, (10)

$$NB_{1}\left(t\right)>NB_{2}\left(t\right)⟺\;s_{1,t}\left({\overset{¯}{Y}}_{1,\geq t}-t\right)>s_{2,t}\left({\overset{¯}{Y}}_{2,\geq t}-t\right)⟺\;\left(1-s_{1,t}\right)\left(t-{\overset{¯}{Y}}_{1,<t}\right)>\left(1-s_{2,t}\right)\left(t-{\overset{¯}{Y}}_{2,<t}\right).$$

That is, higher NB corresponds to a larger above-threshold (or, equivalently, below-threshold) calibration margin, weighted by the fraction treated (or spared).

Furthermore, we note that if $s_{1,t}=s_{2,t},t\in \left(0,1\right)$, then the equivalences in (10) simplify to

$$NB_{1}\left(t\right)>NB_{2}\left(t\right)\;⟺\;{\overset{¯}{Y}}_{1,\geq t}>{\overset{¯}{Y}}_{2,\geq t}\;⟺\;{\overset{¯}{Y}}_{1,<t}<{\overset{¯}{Y}}_{2,<t},$$

which are again the observed event rates from Section “Interpreting net benefit through decision-threshold calibration”.

## Appendix F. Examples for moderate calibration

### F.1. Moderate calibration does not guarantee the same within subgroups

The guarantee of moderate calibration is an overall statement. It does not imply that NB stays above the defaults within every subgroup.

Consider a population consisting of 50 men and 50 women, and suppose the decision threshold is t=0.5. The model assigns only two possible predicted risks, p=0.8 and p=0.2. Let the population be as follows:

$$\begin{array}{cccc} \text{subgroup} & p_{i} & n & q_{i}\\ \text{men} & 0.8 & 10 & 0.4\\ \text{women} & 0.8 & 40 & 0.9\\ \text{men} & 0.2 & 40 & 0.1\\ \text{women} & 0.2 & 10 & 0.6 \end{array}$$

Then the model is moderately calibrated in the overall population, because

$$E\left({\overset{¯}{Y}}_{i:p_{i}=0.8}\right)=\frac{10\cdot 0.4+40\cdot 0.9}{50}=0.8,$$

and

$$E\left({\overset{¯}{Y}}_{i:p_{i}=0.2}\right)=\frac{40\cdot 0.1+10\cdot 0.6}{50}=0.2,$$

using the definition of moderate calibration in Eq. (8).

At threshold t=0.5, all patients with p=0.8 are classified as positive and all patients with p=0.2 as negative. Overall,

$$E\left({\overset{¯}{Y}}_{\geq 0.5}\right)=E\left({\overset{¯}{Y}}_{i:p_{i}=0.8}\right)=0.8>0.5,$$

so the model is better than treat-none. Also,

$$E\left({\overset{¯}{Y}}_{<0.5}\right)=E\left({\overset{¯}{Y}}_{i:p_{i}=0.2}\right)=0.2<0.5,$$

so the model is better than treat-all. Hence NB is in expectation at least as high as both defaults in the overall population.

However, NB falls below a default strategy within both sex subgroups, in different ways. Among men classified positive,

$$E_{men}\left({\overset{¯}{Y}}_{\geq 0.5}\right)=0.4<0.5,$$

so the model is worse than treat-none among men. Among women classified negative,

$$E_{women}\left({\overset{¯}{Y}}_{<0.5}\right)=0.6>0.5,$$

so the model is worse than treat-all among women. Thus, moderate calibration can guarantee NB above the defaults in the target population while masking subgroup-specific failures.

### F.2. Moderate calibration is sufficient but not necessary

Moderate calibration is sufficient, in expectation, for NB at least as high as treat-none and treat-all, but it is not necessary. A model can fail to be moderately calibrated and still have net benefit at least as high as treat-none and treat-all.

Consider a population of 100 patients. The model assigns only two possible predicted risks, $p_{i}=0.8$ and $p_{i}=0.2$, and suppose the true risks are as follows:

$$\begin{array}{ccc} p_{i} & n & q_{i}\\ 0.8 & 50 & 0.9\\ 0.2 & 50 & 0.1 \end{array}$$

This model is not moderately calibrated, because

$$E\left({\overset{¯}{Y}}_{i:p_{i}=0.8}\right)=0.9\neq 0.8,$$

and

$$E\left({\overset{¯}{Y}}_{i:p_{i}=0.2}\right)=0.1\neq 0.2.$$

Thus, the definition of moderate calibration in Eq. (8) is not satisfied.

Nevertheless, NB is not below the default strategies in expectation. For thresholds 0.2<t≤0.8, all patients with $p_{i}=0.8$ are classified as positive and all patients with $p_{i}=0.2$ as negative. Hence

$$E\left({\overset{¯}{Y}}_{\geq t}\right)=E\left({\overset{¯}{Y}}_{i:p_{i}=0.8}\right)=0.9\geq t,$$

so the model is not worse than treat-none. Similarly,

$$E\left({\overset{¯}{Y}}_{<t}\right)=E\left({\overset{¯}{Y}}_{i:p_{i}=0.2}\right)=0.1\leq t,$$

so the model is not worse than treat-all.

For t≤0.2, all patients are classified as positive, so the model coincides with treat-all. In this example, the overall event rate is π=0.5. Hence, for t≤0.2,

$$NB_{\mathrm{all}}\left(t\right)=\frac{\pi -t}{1-t}=\frac{0.5-t}{1-t}\geq 0,$$

so treat-all, and therefore the model, is not worse than treat-none.

For t>0.8, all patients are classified as negative, so the model coincides with treat-none. Since t>0.8>π=0.5,

$$NB_{\mathrm{all}}\left(t\right)=\frac{0.5-t}{1-t}<0,$$

so treat-none, and therefore the model, is not worse than treat-all. Thus, in this example, NB is at least as high as both default strategies across all thresholds, despite the model not being moderately calibrated.
